# A Meta-analysis Reveals S-1-based Chemotherapy Improves the Survival of Patients With Advanced Gastric Cancer

**DOI:** 10.1097/MD.0000000000000652

**Published:** 2015-04-24

**Authors:** Fang-Lan Wu, De-Cheng Lu, Yan-Ping Ying, Jin-Jiao Huang, Ai-Min Zhou, Dun-Ke Jiang, Mao-Wei Chen, Xi Yang, Jia Zhou, Hui-Qiao Huang, Hong-Yan Zeng

**Affiliations:** From the Hospital Quality Management Office (F-LW); Department of Endocrinology (D-CL, A-MZ, XY, JZ, H-QH); Department of Thoracic and Cardiovascular Surgery (Y-PY); Outpatient Department (J-JH, H-YZ); Department of Gastroenterology (D-KJ); and Department of Infectious Disease, First Affiliated Hospital of Guangxi Medical University, Nanning, 530021, Guangxi, China (M-WC).

## Abstract

The aim of this study was to compare the efficacy and safety of S-1-based therapy versus non-S-1-based therapy in advanced gastric cancer (AGC) patients.

Eligible studies stratifying objective response rate (ORR), progression-free survival (PFS), overall survival (OS), and adverse events (AEs) in AGC patients were identified from Embase, Pubmed, Cochrane Library, and China National Knowledge Infrastructure databases. The STATA package (version 11.0) was used to pool the data from the eligible studies.

Fifteen studies with 2973 AGC cases, of which 1497 (50.4%) received S-1-based therapy and 1476 (49.6%) received non-S-1-based therapy, were identified in the meta-analysis. AGC patients who had received S-1-based therapy had a higher median OS, median PFS, and ORR than those who had received 5-fluorouracil (FU)-based therapy (OS: hazard ratio [HR] 0.89, 95% confidence interval [CI] 0.80–0.98, *P* = 0.015; PFS: HR 0.88, 95% CI 0.80–0.98, *P* = 0.016; ORR: OR 1.25, 95% CI 1.08–1.45, *P* = 0.003, respectively). S-1-based therapy had similar efficacy to capecitabine-based therapy in terms of median OS (HR 1.14, 95% CI 0.91–1.41, *P* = 0.253), median PFS (HR 1.01, 95% CI 0.82–1.25, *P* = 0.927), and ORR (OR 0.84, 95% CI 0.63–1.12, *P* = 0.226). Subgroup analysis for grade 3 to 4 toxicity showed higher incidence of neutropenia (relative risk [RR] = 0.827, *P* = 0.006), nausea (RR = 0.808, *P* = 0.040), and lower diarrhea (RR = 1.716, *P* = 0.012) in 5-FU-based arm, and higher diarrhea (RR = 0.386, *P* = 0.007) in capecitabine-based arm.

S-1-based chemotherapy is favorable to AGC patients with better clinical benefit than 5-FU-based chemotherapy and with equivalent antitumor compare with capecitabine-based therapy.

## INTRODUCTION

Gastric cancer is one of the leading causes of death worldwide and prognosis is poor as symptoms often do not appear until the disease has reached an advanced stage.^1,2^ The incidence of gastric cancer is twice as high in men as women and the number of reported cases varies between countries. The number of deaths from gastric cancer has fallen over the past two decades, but it still ranks as the fourth most frequent cancer.^1^ Patients aged 65 years or older account for the most gastric cancer-related deaths.^3^ Although surgery and appropriate adjuvant chemotherapy are used in treatment, the prognosis is poor and average survival is <1 year.^2^

A previous early phase II clinical trial indicated that irinotecan plus cisplatin was beneficial to advanced gastric cancer (AGC) patients, reporting a response rate of 59% and median survival time of 322 days, but with a high incidence of grade 4 neutropenia (57%).^4^ Subsequent work showed that fluorouracil plus cisplatin contributed to a higher response rate and longer progression-free survival. This chemotherapy was used for more toxic events but did not extend survival compared with continuous infusion of fluorouracil alone. Therefore, more effective chemotherapeutic regimens are still required for the treatment of advanced gastric cancer.

In recent years, several phase III studies have been conducted for AGC, using a combination of 5-fluorouracil (5-FU) and cisplatin^5,6^; triple combinations using docetaxel or epirubicin have also been widely tested. A previous clinical trial indicated that irinotecan combined with folinic acid and 5-FU showed similar benefits to epirubicin plus cisplatin and capecitabine, but with a more tolerable toxicity.^7^ A significant improvement in survival was observed when using a combination of docetaxel plus 5-FU plus cisplatin, although the clinical benefit was limited and the regimen affected hematological toxicity.^8^

S-1 is an oral anticancer drug containing tegafur, gimeracil, and oteracil, which has been shown to improve anticancer activity and limit the gastrointestinal toxic effects of FU. The Adjuvant Chemotherapy Trial of S-1 for Gastric Cancer (ACTS-GC) trial indicated that S-1-based therapy prolonged the survival of AGC patients further when compared with surgery alone.^9^ Previous phase II studies reported that S-1 monotherapy was beneficial for AGC patients with a response rate of 45% and a 2-year survival of 17%, with a 5% or lower incidence of grade 3 (or higher) toxic events.^10^ Combinations of S-1 and other cytotoxic drugs, such as docetaxel,^11^ irinotecan,^12^ and cisplatin,^13^ have been explored in several phase I/II studies. With higher objective response rates and lower frequencies of grade 3 or 4 toxic effects, these combinations are thought to be promising. S-1 plus cisplatin has been widely used for the treatment of AGC patients and as the standard chemotherapy regimen for AGC patients in Japan.^14^ This chemotherapy regimen is also being used in the European Union countries (EU) to treat AGC patients.^15^ A meta-analysis showed that S-1-based combination therapy could prolong overall survival (OS), progression-free survival (PFS) and improve objective response rate (ORR) with less toxicity for AGC patients, compared with S-1 monotherapy.^16^ A previous meta-analysis evaluated the efficacy and safety of S-1-based therapy versus 5-FU-based therapy in AGC and reported that S-1-based therapy extended OS with a lower incidence of grade 3 or grade 4 neutropenia, although there was no significant difference in ORR.^17^ This study did not analyze PFS and included only a limited number of eligible studies. Another meta-analysis study, of which 4 randomized controlled trials (RCTs) met our inclusion criteria, reported that S-1-based therapy had more clinical benefits than 5-FU-based therapy; however, the population in this study were all Chinese.^18^ In this study, a meta-analysis was performed on a set of eligible studies to investigate whether S-1-based therapy was more effective than non-S-1-based therapy for treating patients with AGC.

## MATERIALS AND METHODS

### Search Strategy

The Cochrane Library, PubMed, Embase, and China National Knowledge Infrastructure databases were searched to identify all relevant articles published before or on December 2, 2014 using the following key search terms: “S-1,” “Teysuno,” “TS-1,” “tegafur,” “gimeracil,” “oteracil,” “advanced stomach cancer,” “advanced stomach carcinoma,” “advanced gastric cancer,” “advanced gastric carcinoma,” “stomach neoplasm,” and “gastric neoplasm,” “treatment or chemotherapy”. The search was limited to human studies and without language restricted. We also searched for the references of all retrieved studies. In addition, Google Scholar search and all relevant abstracts from the American Society of Clinical Oncology (ASCO) conferences were conducted for supplementation. Finally, we manually selected relevant studies based on the summary analysis. The searches were performed independently by 2 investigators.

### Eligibility Criteria

To be eligible for inclusion in the meta-analysis, all studies had to meet the following criteria: patients had pathologically proven AGC (unrespectable or metastatic) at baseline with no prior radiotherapy or previous adjuvant chemotherapy 1 month before starting the study; compared S-1-based therapy with other agent-based therapies as the first-line chemotherapy regimen; randomized controlled trials or retrospective studies; (4) reported data for calculating the efficacy or safety of these 2 chemotherapy regimens; and (5) presented or allowed the calculation of a hazard ratio (HR) and its 95% confidence intervals (CIs) for OS or PFS compared with 2 chemotherapy regimens. Data from case reports, review articles, and letters were not eligible for our study. When the same patient populations were published in several studies, only the most recent, largest, or complete study was included. Corresponding authors were contacted for more details if necessary. Two independent reviewers assessed all eligible articles using a standardized form.

### Quality Assessment

Two independent reviewers used the Cochrane Handbook for Systematic Reviews of Interventions (version 5.0.2) to assess the quality of the RCTs.^19^ The Newcastle-Ottawa Quality Assessment Scale for cohort studies was used to evaluate the quality of the nonrandomized studies.^20^ Any disagreements were resolved by discussion between the investigators or consulting a third reviewer.

### Data Extraction

The following data were collected from each eligible study: first author, ethnicity, publication year, number of patients evaluated, Eastern Cooperative Oncology Group (ECOG) performance status (PS), proportion of males and average age; (2) study design of the eligible studies, chemotherapy regimen, ORR, median PFS, median OS, and the HR of OS or PFS and its 95% CI; and (3) grade 3 or grade 4 adverse events (AEs). Two reviewers, working independently, used a standardized format to extract the data and this was checked for internal consistency. Consensus were resolved if any disagreement happened.

### Statistical Analysis

The primary endpoints in our study were OS and PFS. The HR and its 95% CI were used to express the association between chemotherapy regimen and the primary endpoints. Either S-1-based therapy results in a shorter PFS or OS, with an HR of more than 1, or it leads to a longer PFS or OS with a HR of <1. The secondary endpoints in our study were ORR and AEs. ORR was defined as the sum of the complete and partial response rates according to the Response Evaluation Criteria in Solid Tumors.^21^ An odds ratio (OR) was used to represent the correlation between the chemotherapy regimen and the ORR of the S-1-based therapy arm over other agent or agent-based combination chemotherapy arms. Thus, there is no significant difference for the ORR between the 2 types of treatment when the OR is equal to 1; a favorable outcome in S-1-based therapy is an OR >1; the tendency of S-1-based patients to be less responsive to treatment is denoted by an OR <1. AEs of the eligible studies were evaluated using the National Cancer Institute's common toxicity criteria (version 2). A *P* value <0.05 was considered statistically significant. A fixed-effects model was conducted to pool HR or OR. We looked for heterogeneity using the traditional Q test and the *I*^2^ index based on standard methods.^22^ The source of heterogeneity was explored using the following techniques: sensitivity analysis, subgroup analysis, or the random-effects model.^23^ Begg test and Egger test were used to look for publication bias.^24,25^ All statistical analyses were performed using the META module of the STATA software program, version 11.0 (Stata Corporation, College Station, TX). A 2-tailed *P* value <0.05 was considered to be statistically significant.

## RESULTS

### Selection of Studies

**FIGURE 1:**
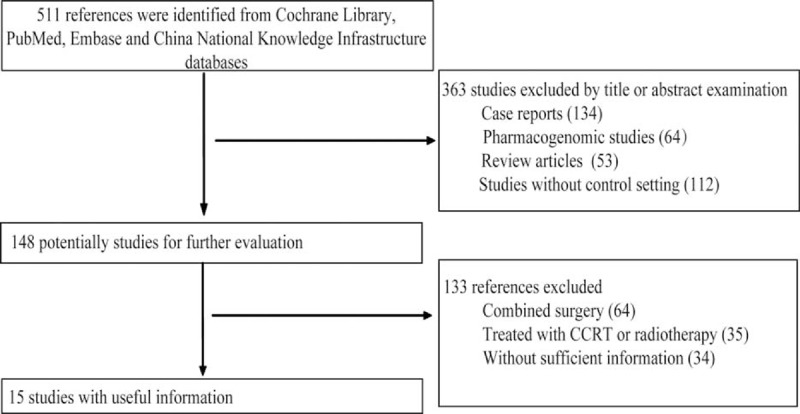
Flowchart of study selection.

A total of 511 related publications were identified based on our initial screening without language restriction. After a careful review of the abstracts, 148 references were deemed eligible based on the inclusion criteria. After reviewing the complete articles, we excluded 64 studies based on the surgical treatment time-period, 35 studies that included concurrent chemo radiotherapy (CCRT) or radiotherapy, and 34 studies with inestimable data or unreachable authors. As a result, the meta-analysis included 15 studies^14,26–39^ involving 2973 AGC patients, with 1497 patients in the S-1-based therapy group (50.4%) and 1476 patients (49.6%) in the non-S-1 therapy group (Fig. 1). Four trail^36–39^ published in Chinese were retrieved from the references of He et al's study.^40^ The characteristics of the 10 included studies are displayed in Table 1.^14,26–39^

**TABLE 1:**
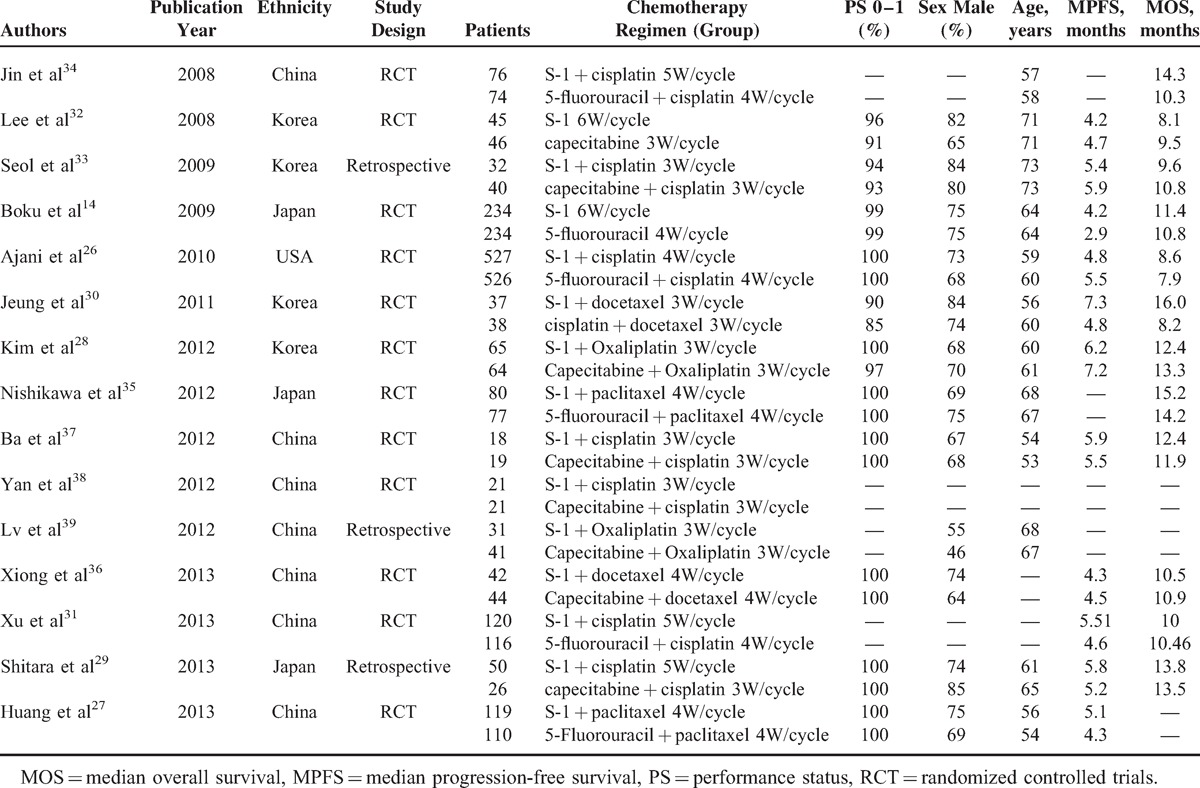
Baseline Characteristics of Eligible Studies

### Quality Assessment of the Studies

We used the Cochrane Handbook for Systematic Reviews of Interventions to assess the quality of the 12 included RCTs and all RCTs reported adequate generation of the allocation sequence. Three studies^14,28,32^ reported allocation concealment (concealed to the investigators). No trials reported a blinding process. Two studies^31,34^ we included are abstract, so the evaluation marks the incomplete outcome, selective reporting, and other bias as unclear. All the RCTs included in our study are level B. The Newcastle-Ottawa Quality Assessment Scale for cohort studies was used to assess the quality of three retrospective studies,^29,33,39^ resulting in high-quality scores with a total of 8 stars. The risk of bias for the 7 RCTs is listed in Table 2.^14,26–28,30–32,34–38^

**TABLE 2:**
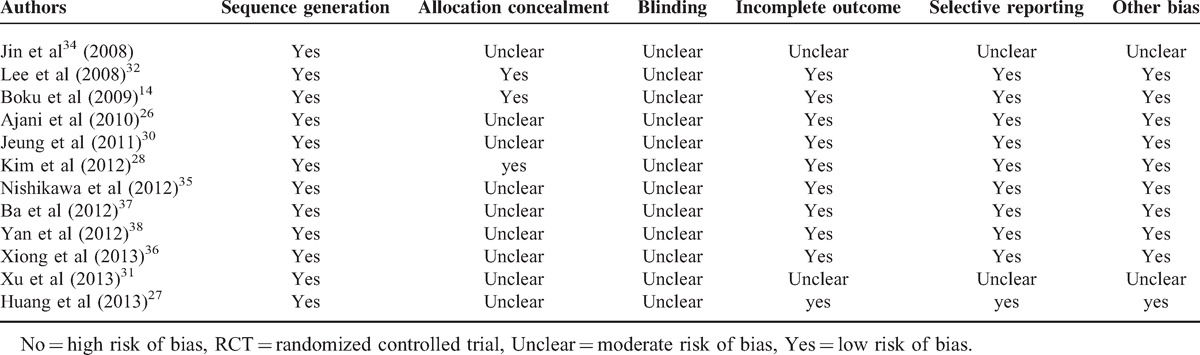
Quality of RCTs Used in the Meta-analysis

### Efficacy

#### Main Results of OS

**FIGURE 2:**
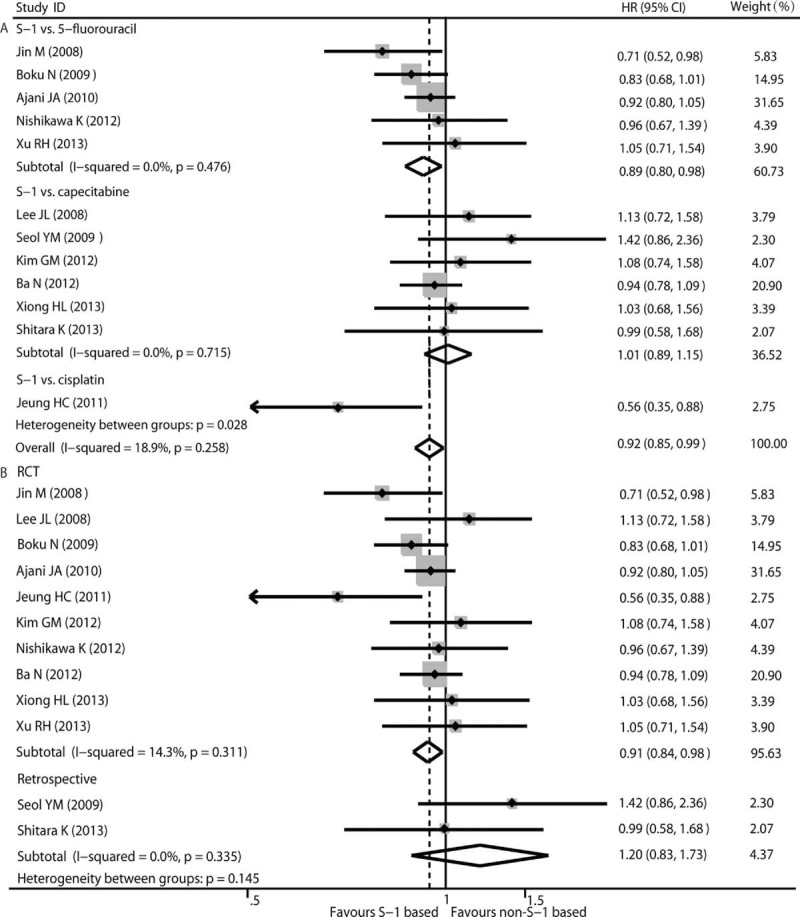
Pooled analyses and subgroup analysis (A, B) of OS associated with S-1-based therapy compared with non-S-1 therapy. HR with its 95% CI <1 indicate a longer OS for S-1 based chemotherapy. HR = hazard ratio, OS = overall survival, RCT = randomized controlled trials.

Twelve eligible studies reported information for treatment and OS; the HR and its 95% CI for OS could be extracted from these studies. Univariate analysis was performed to calculate the HR and the corresponding 95% CI from the references for OS. We looked for some heterogeneity using the Q-test (*χ*^2^ = 13.56, *P* = 0.258, *I*^2^ = 18.9%) with the fixed-effects model. Pooled data from these 12 studies indicated that S-1-based therapy was favorable to AGC patients compared with non-S-1-based therapy (HR 0.92, 95% CI 0.85–0.99, *P* = 0.027) (Fig. 2). Subgroup analysis was performed based on chemotherapy regimen and study design. S-1-based therapy versus 5-FU-based therapy was conducted in 5 studies.^14,26,31,34,35^ The subgroup analysis indicated that AGC patients receiving S-1-based therapy experienced a longer OS compared with patients receiving 5-FU-based therapy (HR 0.89, 95% CI 0.80–0.98, *P* = 0.015) (Fig. 2A). Six studies were performed using capecitabine-based therapy^28–30,33,36,37^ and the subgroup analysis indicated that there was no significant difference in OS benefit (HR 1.01, 95% CI 0.89–1.15, *P* = 0.876) (Fig. 2A). Significant difference between S-1-based arm and non- S-1-based arm also was found in 10 RCTs^14,26,28,30–32,34–37^ (HR 0.91, 95% CI 0.84–0.98, *P* = 0.014) but not in 2 retrospective trials^29,33^ (HR 1.20, 95% CI 0.83–1.73, *P* = 0.336) (Fig. 2B). The results from a sensitivity analysis suggest that our findings are statistically robust. We did not observe any publication bias using either the funnel plot or Egger test (*P* = 0.304 and *P* = 0.587).

#### Main Results of PFS

**FIGURE 3:**
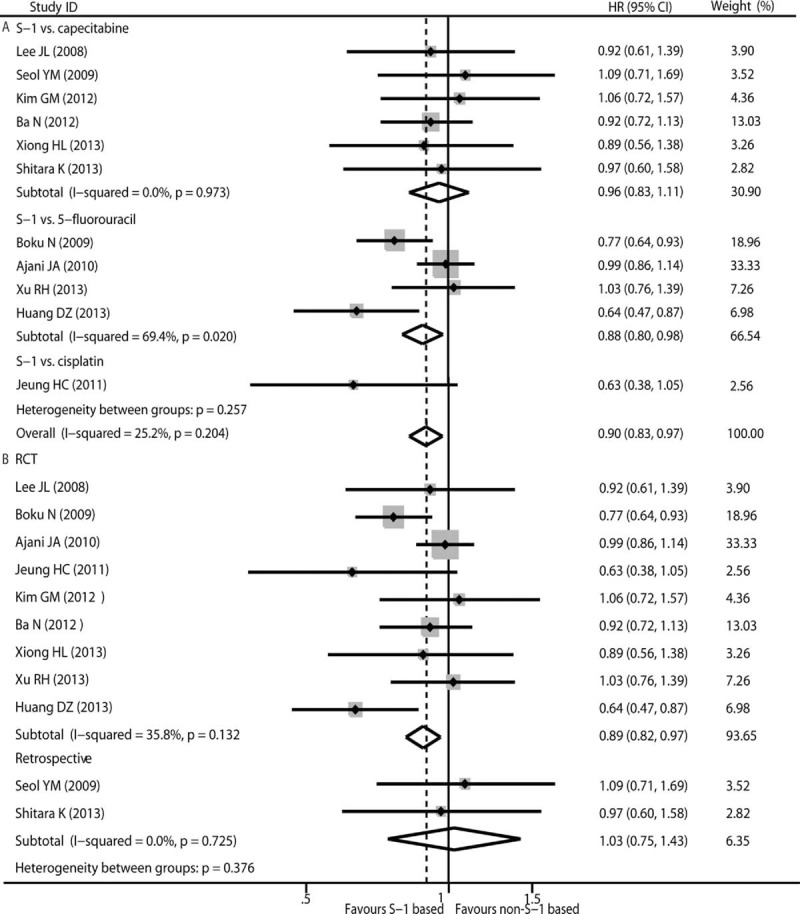
Pooled analyses and subgroup analysis (A, B) of PFS associated with S-1-based therapy compared with non-S-1 therapy. HR with its 95% CI <1 indicate a longer PFS for S-1 based chemotherapy. HR = hazard ratio, PFS = progression-free survival, RCT = randomized controlled trials.

Eleven eligible studies reported the HR with a 95% CI for PFS. The HR and corresponding 95% CI from references for PFS were pooled using univariate analysis. Some heterogeneity was observed using the *Q* test (*χ*^2^ = 13.32, *P* = 0.204, *I*^2^ = 25.2%) with the fixed-effects model. Pooled data of PFS indicated that AGC patients who received S-1-based therapy had a longer PFS than those who received non-S-1-based therapy (HR 0.90, 95% CI 0.83–0.97, *P* = 0.010) (Fig. 3). S-1-based therapy versus 5-FU-based therapy was conducted in 4 studies.^14,26,27,31^ Subgroup analysis with moderate heterogeneity (*χ*^2^ = 9.79, *P* = 0.020, *I*^2^ = 69.4%) indicated that S-1-based therapy showed a favorable outcome for AGC patients by prolonging PFS when compared with 5-FU-based therapy (HR 0.88, 95% CI 0.80–0.98, *P* = 0.016) (Fig. 3A). No heterogeneity was observed in the capecitabine-based therapy group (*χ*^2^ = 0.87, *P* = 0.973, *I*^2^ = 0.00%) and the pooled data indicated that S-1-based and capecitabine-based therapy showed a similar PFS benefit (HR 0.96, 95% CI 0.83–1.11, *P* = 0.567) (Fig. 3A). Significant difference between S-1-based arm and non-S-1-based arm also was found in 9 RCTs^14,26–28,30–32,36,37^ (HR 0.89, 95% CI 0.82–0.97, *P* = 0.007) but not in 2 retrospective trials^29,33^ (HR 1.03, 95% CI 0.75–1.43, P = 0.835) (Fig. 3B). The results from a sensitivity analysis suggest that our findings are statistically robust. No publication bias was detected using either the funnel plot or Egger test (*P* = 0.436 and *P* = 0.719).

#### Overall Response Rate

**FIGURE 4:**
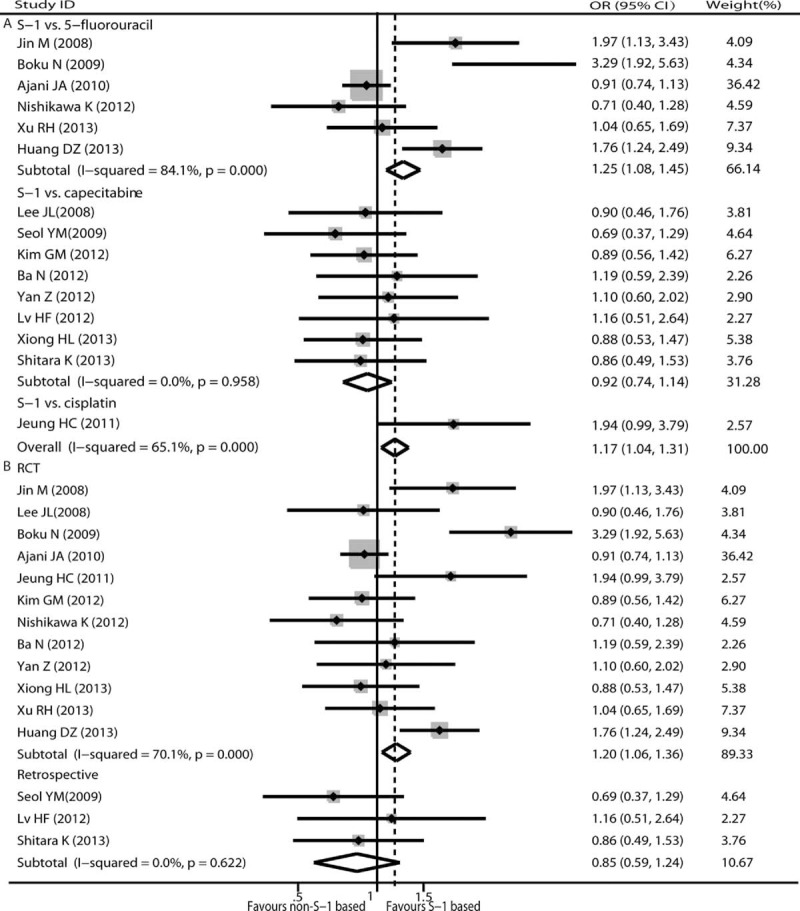
Pooled analyses and subgroup analysis (A, B) of ORR associated with S-1-based therapy compared with non-S-1 therapy. OR with its 95% CI >1 indicates a higher ORR for S-1 based chemotherapy. OR = odds ratio, ORR = objective response rate, RCT = randomized controlled trials.

Tumor objective responses were extracted from all eligible studies, which included 2444 patients. We looked for moderate heterogeneity across studies using the fixed-effects model (*χ*^2^ = 40.12, *P* = 0.00; *I*^2^ = 65.1%), and the subgroup analysis showed that 5-FU-based chemotherapy (*χ*^2^ = 31.36, *P* = 0.00; *I*^2^ = 84.1%) was the main sources of heterogeneity. The ORR of AGC patients with S-1-based therapy was 33.20% (413/1244), whereas that of AGC patients with non-S-1-based regimens was 28.17% (338/1200), which indicated that S-1-based therapy could improve ORR for AGC patients compared with non-S-1-based therapy (OR = 1.17; 95% CI 1.04–1.31; *P* = 0.011) (Fig. 4). In the subgroup analysis of the chemotherapy regimen, the ORR for patients who received S-1-based therapy was higher than that of patients who received 5-FU-based therapy (*P* = 0.003) (Fig. 4A). There was no heterogeneity (*χ*^2^ = 2.02, *P* = 0.958, *I*^2^ = 0.0%) in the capecitabine-based therapy group and the result indicated that capecitabine-based therapy was not superior to S-1-based therapy in overall response rate (*P* = 0.433) (Fig. 4A). Significant difference between S-1-based arm and non-S-1-based arm also was found in 12 RCTs^14,26–28,30–32,34–38^ (HR 1.20, 95% CI 1.06–1.36, *P* = 0.004) but not in 3 retrospective trials^29,33,39^ (HR 0.85, 95% CI 0.59–1.24, *P* = 0.407) (Fig. 4B). The results from a sensitivity analysis suggest that our findings are statistically robust. No publication bias was detected using either the funnel plot or Egger test (*P* = 0.553 and *P* = 0.507).

#### Analysis for non-S1-based Therapy (5-FU vs Capecitabine)

The statistical method published by Altman et al^41^ was used to conducted meta-analysis among non-S1-based therapy, which indicated that similar efficacy of OS (HR 0.88, 95% CI 0.75–1.03) and PFS (HR 1.09, 95% CI 0.91–1.30) was found between 5-FU-based arm and capecitabine-based arm other than capecitabine-based arm had higher ORR (relative risk [RR] 1.36, 95% CI 1.05–1.76).

#### Toxicity

The toxicity profile analyses for eligible trials are shown in Table 3. The most common grade 3 to 4 hematologic toxicities were neutropenia and anemia in both arms, and the most frequent grade 3 to 4 nonhematological toxicities were nausea for each arm. Pooled data revealed no significant difference in safety profiles for both grade 3 to 4 hematologic and grade 3 to 4 nonhematological events, other than lower incidence of neutropenia and vomiting were observed in S-1-based arm. Subgroup analysis for grade 3 to 4 toxicity showed higher incidence of neutropenia (RR = 0.827, *P* = 0.006) and nausea (RR = 0.808, *P* = 0.040), lower diarrhea (RR = 1.716, *P* = 0.012) in 5-FU-based arm, and higher diarrhea (RR = 0.386, *P* = 0.007) in capecitabine-based arm. Analysis among non-S1-based arm, which was performed via the method of Altman et al literature,^41^ showed that 5-FU-based arm had lower incidence of diarrhea than that in capecitabine-based arm (RR 4.45, 95% CI 1.98–9.98) (Table 4).

**TABLE 3:**
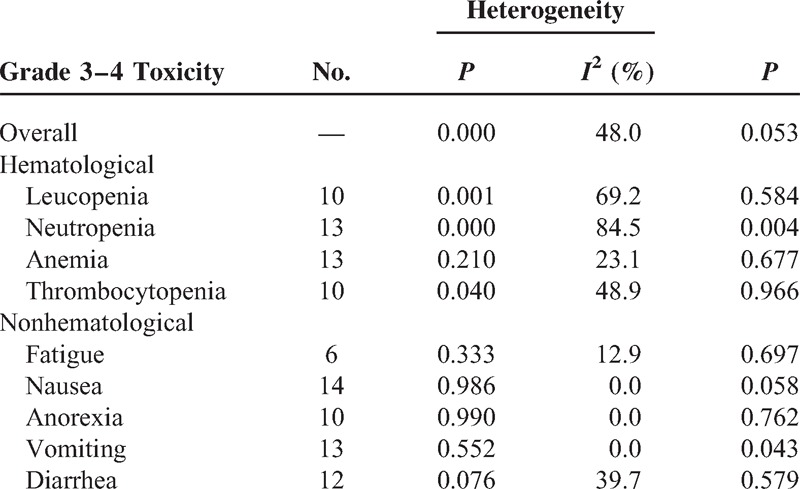
Outcome of Grade 3–4 Toxicity Meta-analysis

**TABLE 4:**
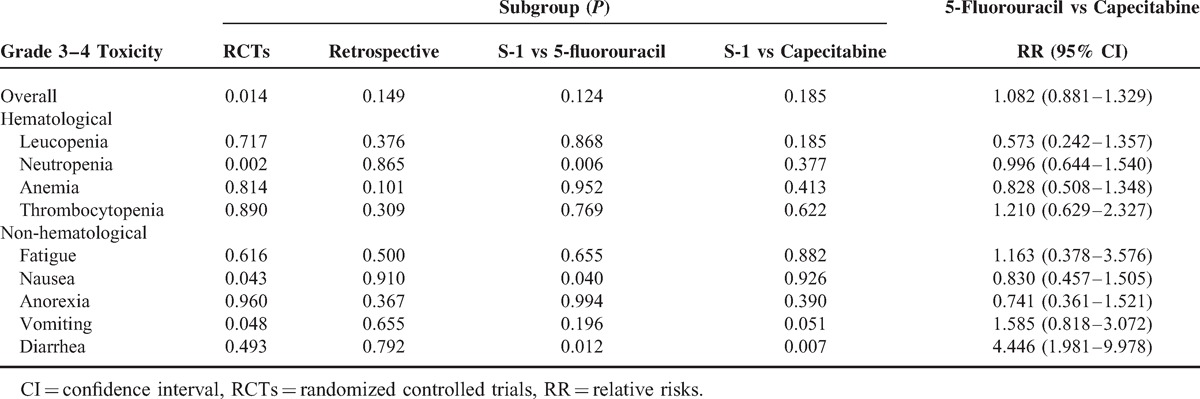
Subgroup Analyses for Grade 3–4 Toxicity

## DISCUSSION

Several early phase II and III studies have reported that S-1 has a multifactorial synergistic effect on anti-tumor activity in AGC patients in a single agent setting.^10,42^ In addition, several trials exploring combinations of S-1 and other agents for the treatment of AGC have been conducted widely in Asian countries including Japan, China, and Korea. However, as yet there is no consensus in the medical community regarding the superiority of S-1-based therapy over other agent or agent-based therapies for AGC patients. The meta-analysis of published data presented in this study aimed to address this by determining whether S-1-based therapy could improve objective response rate, control symptoms, prolong survival, and maintain or improve quality of life compared with non-S-1-based therapy in patients with AGC.

The results from our study indicate that S-1-based chemotherapy showed greater clinical benefit in terms of OS, PFS, and ORR; achieved tolerability 3 to 4 toxicity for AGC patients when compared with 5-FU-based therapy, and has similar clinical benefit with capecitabine-based therapy. In the subgroup analysis of the chemotherapy regimen for OS, S-1-based therapy prolonged the OS in AGC patients compared with 5-FU-based therapy. This finding concurred with a previous meta-analysis conducted by Huang et al.^17^ We note that Fuse et al's study^43^ is the updated analysis of Boku et al's study^14^ with the same patients population and both of these studies were included in the Huang et al's study^17^; with careful consideration, the study conducted by Fuse et al^43^ was excluded from our study because of insufficient information. Furthermore, the results in our study were generated from a larger sample size and a greater number of studies; therefore it could be considered more statistically robust than the aforementioned study.^17^

When S-1-based therapy was compared with 5-FU-based therapy with regard to PFS, the former was found to be more effective (*P* = 0.016) for AGC patients. This particular finding was not reported in the previous meta-analysis by Huang et al.^17^ With regard to the ORR, the Huang et al's study^17^ reported that S-1-based therapy was not superior to 5-FU-based therapy (OR = 1.25, 95% CI 0.31–5.09, *P* = 0.734). However, our results from the subgroup analysis indicated the contrary. The reasons for this might be that only 2 studies provided relevant information of overall response rate in the Huang et al's study,^17^ whereas we found 6 such studies. Furthermore, the data extracted from the trial by Boku et al's study^14^ regarding the ORR in the 5-FU-based therapy group that is referenced in the Huang^17^ study should be 15/175, not 68/181. Another meta-analysis,^44^ which included the same 6 articles with our current analysis, used randomized-effects model to pool OR for ORR among S-1-based and 5-FU-based because high heterogeneity was observed, with the results that no significant difference was found in terms of ORR between these 2 arms. It seems that the total studies size of six might not warrant using randomized-effects model; therefore, fixed-effects model was conducted and the result indicated that S-1-based arm had higher ORR than the 5-FU-based arm. This result was consistent with Yang et al's.^45^ The purpose of our study is consistent with Yang et al’ study^45^ with similar conclusions, but more related RCTs were included in our current trail.

There was insufficient information on safety profiles in 1 eligible study,^30^ even after contacting the author. The pooled data on toxic effects indicated no significant difference in most of grade 3 to 4 toxic events between S-1-based therapy and non-S-1-based therapy, and all the toxicities were manageable, tolerable, and predictable. Although high heterogeneity was found in subgroup analysis for grade 3 to 4 neutropenia, the main sources of heterogeneity came from 5-FU-based chemotherapy group including 6 literatures; therefore, we still used fixed-effects model to perform analysis.

Two previous meta-analysis articles^40,46^ showed that S-1-based treatment had similar antitumor efficacy with capecitabine-based treatment for AGC patients; 4 trials included in them were not included in our current analysis because those 4 trails clearly stated that S-1 provided to the AGC patients was made in china, which might lead to clinical heterogeneity. Conclusions from other meta-analysis studies have indicated that S-1-based and capecitabine-based chemotherapy treatments are similarly effective and well tolerated for gastrointestinal cancers^47^; in this case, our work is limited to advanced gastric cancer; therefore, 2 studies related to colorectal cancer were excluded from our work. We noted that the conclusion we drew from the capecitabine-based therapy subgroup analysis is similar to that of the 3 meta-analysis studies mentioned above.

There are some clear weaknesses in our study. First, we used the method posed by Altman et al^41^ to assess the efficacy and tolerability of non-S-1-based chemotherapy among AGC patients, and our results showed that higher ORR and grade 3 to 4 diarrhea were observed in capecitabine-based arm compared with 5-fluorouracil-based arm. This conclusion needs to be confirmed because these calculations are used for comparing 2 estimated RRs from subgroup analysis. Second, the eligible studies included 12 RCTs with level B in the quality assessment and three studies^29,33,39^ with higher score, and the results could have been affected by the quality of the individual studies. Finally, most of the studies in our analysis comprised populations from East Asia who have a similar genetic background; only 1 study was conducted in the West (USA).^26^ Therefore, we did not perform a subgroup analysis based on ethnicity, and these conclusions should be confirmed via high-quality RCTs and Western studies.

Overall, our study shows that S-1-based chemotherapy is favorable to AGC patients with better clinical benefit in term of OS, PFS, and ORR when compared with 5-FU-based chemotherapy and have similar antitumor efficacy compared with capecitabine-based chemotherapy. Financially, S-1 is cheaper than continuous infusion of fluorouracil and it benefits from the convenience of oral administration. We would therefore recommend S-1-based therapy as a chemotherapeutic regimen for AGC patients in future, once our findings have been confirmed through larger studies and further clinical trials.
